# Mediating Effects of Adolescent Physical Activity, Self-Rated Health and Family Income

**DOI:** 10.3389/fpubh.2022.940141

**Published:** 2022-06-22

**Authors:** Meng Wang, Xi Shen, Lamei Deng, Feng Yu, Yin Lou, JunJi Liu, Yibing Huang

**Affiliations:** ^1^Department of Health Education, Hangzhou Center for Disease Control and Prevention, Hangzhou, China; ^2^Department of Student Manager and Education, Hangzhou Medical College, Hangzhou, China

**Keywords:** family income, physical activity, self-rated health, teenagers, mediation

## Abstract

**Objective:**

To investigate and analyse the situation and relationship between adolescent physical activity and self-assessment of health to provide a reference for adolescent physical activity research.

**Methods:**

A questionnaire was used to investigate the physical activity and self-rated health of 1,804 adolescents aged 14–18 years.

**Results:**

There was a significant relationship between adolescents' physical activity and self-rated health. The coefficient was 0.109 (*P* < 0.01) in urban areas and 0.127 (*P* < 0.01) in rural areas. At the same time, it was found that when family income was used as the intermediary variable between physical activity and self-rated health, the intermediary effect was 0.12 (*P* < 0.01), and the intermediary effect accounted for 25.97%.

**Conclusion:**

Adolescent obesity, physical activity, smoking, wellbeing and physical activity can affect adolescents' self-rated health status. At the same time, it is also found that adolescents' family income is an intermediary variable between physical activity and self-rated health.

**Suggestions:**

(1) Increase the methods of sports venues, sports organizations and sports activities, improve the possibility of teenagers participating in physical activities, and improve teenagers' self-rated health; (2) There is a large gap between the physical activity and self-rated health of urban and rural adolescents. Increasing the guidance of physical activity of adolescents in rural areas promotes the balance of self-rated health of urban and rural adolescents. (3) Family income is the intermediary variable of teenagers' physical activities and self-rated health. Reducing family expenditure through financial transfer payments or reducing taxes and fees can increase the level of teenagers' physical and mental health.

## Introduction

The majority of young people are healthy in body and mind, strong in physique, strong in will and full of vitality. They are the embodiment of the vigorous vitality of a nation, the symbol of social civilization and progress, and an important aspect of the country's comprehensive strength. China attaches great importance to the healthy growth of young people. Since the reform and opening up, China's youth sports have developed vigorously, great achievements have been made in school sports, and the nutritional level and morphological development level of teenagers have been continuously improved, which has greatly improved the health quality of the whole population. However, we must be soberly aware that, on the one hand, due to the influence of the one-sided pursuit of the enrollment rate, society and schools tend to pay more attention to intellectual education and less attention to physical education, students' schoolwork burden is too heavy, and the time for rest and exercise is seriously insufficient; on the other hand, due to the lack of sports facilities and conditions, students' physical education and physical activities are difficult to ensure. Recent physical health monitoring shows that physical indicators such as endurance, strength and speed of adolescents continue to decline, the rate of poor vision remains high, the proportion of overweight and obese adolescents in cities has increased significantly, and the nutritional status of some rural adolescents needs to be improved (1). If these problems are not effectively solved, they will seriously affect the healthy growth of teenagers. In today's China, family income determines the class status of parents, while parents with higher socioeconomic status rely on the advantages of various capital stocks to enable their children to obtain better physical activity opportunities and under the combined action of other factors (education, opportunities, talents, etc.,), build their children's sports ability, and promote the transformation of this sports ability into intangible health capital to form cumulative advantages in the process of growth (2). It helps to obtain more favorable social incentives and resources. Early health status and exercise habits are closely related to class reproduction and social mobility. Therefore, family income will play an important role in teenagers' physical activities and self-rated health.

A large number of facts at all times and in all countries have proven that health and physical activity are not only a pure medical problem but also closely related to many social factors. At the same time, they follow the obvious law of social distribution (3). For example, people have long realized that disease and poverty are like twin brothers. In almost all plague outbreaks recorded in human history, the impact of the poor is much higher than that of the rich, and the prevalence and mortality of the former are often higher than those of the latter. That is, the slight improvement of any social class corresponds to the continuous improvement of the average health status of the corresponding group. A typical example is that even in extremely wealthy Hollywood, there are obvious health differences. Those who have won Oscars live longer on average than those who have only won nominations, and the life expectancy of the former is as much as 4 years higher than that of the latter (4). This phenomenon is called the “social gradient on health,” which is common in almost all societies. Therefore, there is a phenomenon of social stratification in health, and social stratification will also have different reflection and action mechanisms in different groups.

The adolescent stage is a period when individuals change their lifestyle from children to adults, and their behavior has strong plasticity (5). Effective behavioral interventions in this period can help teenagers develop a healthy and active lifestyle. In fact, the existing research in the field of physical activity often focuses on the adult stage or special population stage, and there is less research on teenagers' physical activity behavior.

At present, most studies have verified the positive relationship between physical activity and self-rated health. For example, a study on 15 EU countries shows that physical activity is significantly related to better self-assessment of health, and self-assessment of health is related to age, gender, and income education level (6). Some studies have also found that physical activity in leisure time is positively correlated with self-rated health, and drinking has nothing to do with self-rated poor health (7). At the same time, some studies have found that the total time of physical activities, such as other jobs, cannot replace physical activities (8). The self-assessment of the health of active people who do not exercise and maintain other physical activities for more than 2 h a day is lower (9). At the same time, some studies believe that physical activity can increase people's subjective identity, and the improvement of subjective identity requires a certain income base. Families with higher income will improve their health level through their economic capital (10). This is because family income, as a type of social capital, can affect teenagers' physical and mental health through a variety of ways or channels. Among them, the explanation is more fully understood as follows: first, family income helps teenagers have more opportunities to understand the importance of physical and mental health and the ability to further obtain relevant knowledge, which helps individuals better adjust their own lifestyle and habits to achieve the purpose of physical and mental health; second, teenagers with higher family income can more easily obtain cultural capital and social resources and optimize conditions by improving their material ability and choosing the environment to achieve the purpose of health (11); third, more family resources can also enhance and improve individual social relations and non-material resources of social support. Groups with relatively high cultural resources often have significant advantages in population quality, marital relations and peer resources among groups. This advantage also helps individuals improve their lifestyle and achieve physical and mental health. Fourth, teenagers with higher family income often receive better education. They may have better self-control ability and correct value orientation, which makes this group pay more attention to the meaning of life to choose a good lifestyle, which promotes the improvement of health.

From the current research situation, there are relatively few empirical studies on whether teenagers' physical activities can affect teenagers' self-rated health status, while research using family income as an intermediary variable has not yet appeared. Therefore, this paper will focus on two issues: empirical research on adolescents' physical activity and self-rated health status and the mediating effect of family income as an intermediary variable.

## Methods

This study considered the relationship between adolescents' physical activity and self-rated health by establishing a regression equation. Generally, individual gender, years of education, family income, smoking, obesity and other variables are more important variable factors affecting adolescents' participation in physical activities. Therefore, in constructing the regression equation, this study included the above variables in the variables for investigation. At the same time, considering China's urban-rural dual structure system, we interpret the urban-rural model separately in the regression model.

In the design of the research model, this paper takes two steps: the first step is to use the logistic model for regression fitting analysis; the second step is to conduct an intermediary test with KHB. Similar methods are often used in computational sociology (12, 13).

From February to March 2020, 1,940 questionnaires were collected from students in Shanghai, Anhui, Zhejiang and Jiangsu, China. Because the online questionnaire cannot face the respondents, to improve the accuracy of the questionnaire, the study only analyses the samples aged 14–18 years. The questionnaire completion time was <40 s, and missing data and invalid questionnaires were deleted. After the data of each variable are cleared, the actual sample size used in the study is 1,804. To ensure the reliability of the questionnaire, in this study, we measured some young students in the first questionnaire again after 2 weeks and calculated the reliability coefficient of the second measurement as *R* = 0.82 (*P* < 0.05), indicating that the index system of the questionnaire is reliable.

### Dependent Variable: Self-Rated Health

Self-rated health is obtained by answering the self-health status of adolescents in the past 6 months. The questionnaire adopts a five-level scale: very bad, bad, average, good and very good. In the analysis of this paper, the continuous variables of 1–5 are transformed according to the number of frequencies.

### Independent Variable

Exercise frequency, that is, the average number of physical exercises per week. Exercise frequency is a categorical variable. 1 represents “less than once a week,” 2 represents “1–2 times a week,” 3 represents “3–4 times a week”, 4 represents “5 times a week,” 5 represents “once a day,” and 6 represents “twice a day.”

### Family Income

According to the content of the questionnaire, this paper uses annual family income as the measurement variable and takes the logarithm of family income for processing.

### Other Variables

The factors affecting health are complex. In addition to the common gender and age variables, the study mainly includes whether smoking and obesity affect health in daily life into the control variables. The occasional and small amount of smoking is treated as non-smoking, which is set as 0 = smoking and 1 = non-smoking. An obesity index (BMI) ≥ 29 and <30 is called an obesity index 9, BMI ≥25 is called overweight, BMI <24 9 and BMI ≥18 5 is called normal, and BMI <18 5 is called lean. Obesity variables are regarded as categorical variables: “1” represents lean, “2” represents normal, “3” represents overweight, and “4” represents obesity. At the same time, we take happiness as one of the variables because happiness can improve individual health. In the questionnaire, we divide happiness into five grades: “1” represents very unhappy, “2” unhappy, “3” normal, “4” relatively happy, and “5” very happy. According to the urban-rural situation, China's urban-rural dual system structure makes the gap between urban and rural areas large. Therefore, this paper divides the research object into urban and rural groups, in which rural = 0 and urban = 1. See Table 1 for details.

**Table 1:** Descriptive statistics.

**Variable**	**Obs**	**Mean**	**Std. dev**.	**Min**	**Max**
Self-rated health	1,804	3.131	1.589	1	5
Gender	1,804	0.513	0.499	0	1
Physical activity frequency	1,804	2.571	0.891	1	6
Family income	1,804	2.207	0.877	−2.303	6.215
Obesity	1,804	1.932	0.751	1	4
Do you smoke	1,804	0.562	0.471	0	1
Happiness	1,804	3.158	1.224	1	5
Age	1,804	16.243	0.892	14	18

## Main Results

Table 2 shows the regression results of three logistic models.

**Table 2:** Regression model of physical activity and self-rated health identity of Chinese adolescents.

**Variable**	**Comprehensive model**	**City model**	**Rural model**
	**Model 1**	**Model 2**	**Model 3**
Urban and rural	0.481^***^ (0.088)		
Gender	0.007 (0.065)	0.073 (0.069)	−0.322^**^ (0.147)
Physical activity frequency	0.121^***^ (0.012)	0.109^***^ (0.022)	0.127^***^ (0.021)
Age	−0.015 (0.02)	−0.021(0.023)	0.026 (0.065)
Happiness	0.004^***^ (0.024)	0.005^***^ (0.016)	0.007^***^ (0.048)
Obesity	−0.111^***^ (0.037)	−0.086^**^ (0.031)	−0.131 (0.087)
Do you smoke	0.378^***^ (0.112)	0.388^***^ (0.102)	0.335 (0.251)
Family income	0.771^***^ (0.154)	0.742^***^ (0.181)	0.981^**^ (0.441)
_cons	−2.513^***^ (0.276)	−1.99^***^ (0.321)	−2.499^***^ (0.67)
Observations	1,804	1,002	802
Pseudo R^2^	0.073	0.059	0.062

Standard errors are in parentheses. ^***^p < 0.01, ^**^p < 0.05, ^*^p < 0.1.

Model 1 is a comprehensive regression model including urban and rural variables, that is, the overall social adolescent physical activity and self-rated health model. In model 1, there was a significant relationship between adolescent physical activity and self-rated health, and the coefficient was 0.121 (*P* < 0.01). There was a significant relationship between urban and rural variables of adolescents' residence and self-rated health, and the coefficient was 0.481 (*P* < 0.01). There was a significant relationship between adolescents' wellbeing and self-rated health (*P* < 0.01). There was a significant relationship between obesity and self-rated health in adolescents, and the coefficient was −0.111 (*P* < 0.01). There was a significant relationship between smoking and self-rated health in adolescents, and the coefficient was 0.378 (*P* < 0.01). There was a significant relationship between adolescent family income and self-rated health, and the coefficient was 0.771 (*P* < 0.01).

Model 2 is the regression model of physical activity and self-rated health status of adolescents with urban variables, that is, the model of physical activity and self-rated health of adolescents living in urban communities. In model 2, there was a significant relationship between adolescent physical activity and self-rated health, and the coefficient was 0.109 (*P* < 0.01). There was a significant relationship between adolescents' wellbeing and self-rated health (*P* < 0.01). There was a significant relationship between obesity and self-rated health in adolescents, and the coefficient was −0.086 (*P* < 0.01). There was a significant relationship between smoking and self-rated health in adolescents, and the coefficient was 0.388 (*P* < 0.01). There was a significant relationship between adolescent family income and self-rated health, and the coefficient was 0.742 (*P* < 0.01).

Model 3 is the regression model of physical activity and the self-rated health status of adolescents in rural areas, that is, the model of physical activity and the self-rated health of adolescents living in rural areas. In model 3, there was a negative correlation between adolescents' gender and the self-rated health model, and the regression coefficient was −0.322 (*P* < 0.05). There was a significant relationship between adolescents' physical activities and self-rated health, and the coefficient was 0.127 (*P* < 0.01); there was a significant relationship between self-evaluation of wellbeing and adolescent health (*P* < 0.01); there was a significant relationship between obesity and self-rated health in adolescents, and the coefficient was −0.131 (*P* < 0.01); there was a significant relationship between smoking and self-rated health in adolescents, and the coefficient was 0.335 (*P* < 0.01); and there was a significant relationship between adolescent family income and self-rated health, and the coefficient was 0.981 (*P* < 0.01).

Table 3 shows the analysis results of the mediating effect between physical activity and the self-rated health of adolescents. The total effect of physical activity on the self-rated health of adolescents is 0.462 (*P* < 0.01), and the mediating effect of family income as the mediating variable is 0.12 (*P* < 0.01), accounting for 25.97%.

**Table 3:** The intermediary effect of adolescent self-assessment and physical activity.

**Intermediary variable**	**Independent variable**	**Total effect**	**Direct effect**	**Intermediary effect**	**Proportion of intermediary effect**
Family income	Physical activity frequency	0.462^***^	0.342^***^	0.12^***^	25.97%

Standard errors are in parentheses. ^***^p < 0.01, ^**^p < 0.05, ^*^p < 0.1.

## Discussion

Self-rated health is the comprehensive subjective feeling of the respondents on all aspects of their own health status. It can effectively reflect the subjective and objective aspects of health status. In particular, this subjective feeling is based on objective health status and can comprehensively reflect the related functions of physical health, mental health and social role. Teenagers' response to self-assessment of health not only has the response of physical health but also receives the influence of social psychology. In this empirical study, there are urban-rural differences in the impact of Chinese teenagers' health awareness. The self-rated health of teenagers living in cities is better than that of teenagers living in rural areas, which shows that under China's urban-rural dual system structure, the difference in social resources not only affects the difference in economic and social development but also affects the difference in teenagers' healthy development. There are almost no gender differences in adolescents' self-rated health in cities, while in rural areas, male adolescents have better self-rated health than female adolescents, which also reflects that the living conditions of female adolescents in rural areas are relatively poor, which is related to the concept of men being superior to women in rural areas of China; that is, in rural areas of China, women often undertake housework from an early age. At the same time, the idea of son preference makes women in rural areas more likely to choose to watch TV and other activities at home (10, 14). It was found that the self-assessment activities of adolescents in rural areas can promote their physical health. At the same time, the self-assessment activities of adolescents in rural areas can also play a positive role in promoting their physical health, which also shows that teenagers in China's vast rural areas need to improve their physical and mental health through physical activities. There is a basic consensus that adolescents' subjective wellbeing improves their self-rated health. After all, subjective wellbeing improves adolescents' physical and mental pleasure. However, in this study, it is found that compared with urban areas, the improvement of adolescents' wellbeing in rural areas makes it easier to improve their self-rated health level than that in urban areas, which also reflects the relatively simple lifestyle and content in rural areas. Often, some resources other than necessary living resources are obtained, which will bring greater marginal benefits. In the national model and urban model, there is a negative correlation between obesity and self-rated health awareness, while there is no significant relationship between obesity and health awareness in rural areas, which shows that adolescents in urban areas are better than adolescents in rural areas in terms of physical health awareness, while adolescents in rural areas also lack obesity and self-rated health awareness due to their backwardness in social development, economy and education. Whether in urban or rural areas, this study found a positive correlation between family income and self-rated health, which is similar to previous research results. In other words, income will affect the nutritional status of adolescents in the early stage of life, the life accumulation of various favorable or unfavorable factors with increasing age, the availability of medical security resources, and the right to choose a healthy lifestyle given by income and wealth. This study also found that adolescents with higher family income have better health status, which is particularly prominent among rural adolescents.

In fact, as an intermediary factor between physical activity and self-rated health, family income explains 25.97% of the possibility. This shows that the increase in income is not an absolute value but also an important coordinate of relative position. Although according to the relevant theories of economics, the relative position of individuals in society can lead to important psychosocial effects, adolescents with relatively low social status will produce negative psychosocial factors such as tension, anxiety, stress, despair and out of control, which are likely to cause health problems. Family income acts as an intermediary variable between physical activity and health. On the one hand, teenagers often participate in physical activities with their parents or guided by their parents, which improves the social capital of the family through the participation of physical activities. The increase in family social capital provides material and spiritual protection for teenagers' physical and mental health (15).

## Conclusion

Through the survey data of adolescents in three provinces and one city, using a regression model and KHB intermediary analysis, this paper finds that adolescents' obesity, physical activity, smoking, wellbeing and physical activity affect adolescents' self-rated health status. At the same time, it also finds that adolescents' family income is the intermediary variable of physical activity and self-rated health. In view of the above findings, to better guide teenagers' physical activities and improve their self-rated health level, this study puts forward the following suggestions: (1) Increase the methods of sports venues, sports organizations and sports activities, improve the possibility of teenagers' participation in physical activities and improve teenagers' self-rated health; (2) There is a large gap between physical activity and the self-rated health of urban and rural adolescents. Increasing the guidance of physical activity of adolescents in rural areas promotes the balance of self-rated health of urban and rural adolescents. (3) Family income is the intermediary variable of teenagers' physical activities and self-rated health. Reducing family expenditure through financial transfer payments or reducing taxes and fees can increase the level of teenagers' physical and mental health.

## Research Advantages and Disadvantages

Using empirical research data, this paper reveals the related influencing factors and mediating mechanism of adolescents' physical activity and self-rated health in the Yangtze River Delta of China. It can improve the coverage and effectiveness of adolescent self-assessment data. However, due to the large differences between urban and rural areas, North–South differences and East–West differences in China, the research in this paper has not covered different regions for investigation and discussion. Therefore, there are some regrets. In the follow-up, we can continue to carry out relevant research on teenagers in different regions.

## Data Availability Statement

The raw data supporting the conclusions of this article will be made available by the authors, without undue reservation.

## Ethics Statement

The studies involving human participants were reviewed and approved by Anhui Professional & Technical Institute of Athletics. Written informed consent from the participants' legal guardian/next of kin was not required to participate in this study in accordance with the national legislation and the institutional requirements.

## Author Contributions

MW and LD drafted the work. LD, FY, and YL contributed to the initial drafting of the manuscript. XS revised and improved the article. All authors contributed to the article and approved the submitted version.

## Conflict of Interest

The authors declare that the research was conducted in the absence of any commercial or financial relationships that could be construed as a potential conflict of interest.

## Publisher's Note

All claims expressed in this article are solely those of the authors and do not necessarily represent those of their affiliated organizations, or those of the publisher, the editors and the reviewers. Any product that may be evaluated in this article, or claim that may be made by its manufacturer, is not guaranteed or endorsed by the publisher.
